# How does mindful awareness impact academic performance in junior high school students? A chain mediation effect based on academic self-efficacy and academic buoyancy

**DOI:** 10.3389/fpsyg.2025.1687223

**Published:** 2025-11-11

**Authors:** Jianwen Guo, Jiahao Zhang, Lei Qiao, Yun Zhou

**Affiliations:** 1 College of Elementary Education, Capital Normal University, Beijing, China; 2 School of Education, Minzu University of China, Beijing, China; 3 Academic Affairs Office, Pu’er University, Puer, China; 4 Teaching Research Institute, Teaching Affairs Office, Northern Nationalities University, Yinchuan, China

**Keywords:** mindful awareness, academic performance, junior high school students, academic self-efficacy, academic buoyancy

## Abstract

**Background:**

Previous research has mostly focused on exploring the mechanisms of mindful awareness and academic performance in university students, with less attention paid to junior high school student group. This study aimed to examine how mindful awareness influences academic performance in junior high school students through the chain mediation of academic self-efficacy and buoyancy.

**Methods:**

The study selected 3,163 Chinese junior high school students (1,599 males, 1,564 females; mean age = 13.30, standard deviation = 0.90), and assessed their mindful awareness, academic buoyancy, academic self-efficacy, and academic performance through a survey.

**Results:**

The results showed that mindful awareness had a significant positive effect on academic performance (β = 0.179). This relationship was partially mediated through two pathways: academic self-efficacy (β = 0.083) and academic buoyancy (β = 0.035). Additionally, a serial mediation pathway through both mediators was significant (β = 0.024). These indirect effects collectively accounted for 44.2% of the total effect, suggesting that mindful awareness enhances academic performance both directly and indirectly through increased academic self-efficacy and buoyancy.

**Conclusion:**

The research findings provide a theoretical and practical basis for enhancing students’ positive psychology and promoting academic performance through mindful awareness interventions. These findings suggest that schools could improve student outcomes by integrating mindfulness practices (e.g., brief pre-class meditation) into support programs.

## 1 Introduction

Mindfulness was initially introduced into psychology as an intervention approach, the essence of which involves consciously directing attention to present-moment internal and external experiences through practices such as meditation (Bishop et al., 2004). In recent years, mindful awareness has been increasingly applied in educational contexts (e.g., Monsillion et al., 2023; Ostermann et al., 2022; Phillips and Mychailyszyn, 2022; Marshall et al., 2022), demonstrating substantial benefits for enhancing students’ academic performance. Specifically, students with higher levels of mindful awareness tend to achieve superior academic outcomes (Vorontsova-Wenger et al., 2021). Although existing empirical evidence indicates a significant positive association between mindful awareness and academic performance, the underlying mechanisms remain inadequately understood and warrant further investigation. Research suggests that two core effects of mindfulness training involve enhanced attention and cognitive control, as well as improved emotion regulation capacity (Charness et al., 2024; Yavuz Sercekman, 2024). This suggests that mindful awareness may influence psychological adaptation through improved cognitive and emotional functioning, thereby serving as a potential contributor to academic performance.

Social cognitive theory posits that physiological and emotional states constitute critical factors influencing the formation of individual self-efficacy. Negative emotional states such as stress lead individuals to underestimate their capabilities, thereby exerting adverse effects on self-efficacy (Liu et al., 2024). Mindful awareness is typically defined as intentional, non-judgmental attention to present-moment experiences (Bishop et al., 2004). Through facilitating decentering, it enables individuals to reappraise stressful events and negative emotions as transient mental states rather than self-invested authentic experiences, thereby down-regulating the processing of stressful events and emotional responses (Lebois et al., 2015). This suggests that mindful awareness can mitigate psychological stress perception by enhancing individuals’ emotional awareness and regulation capacity (Bao et al., 2015), consequently buffering the negative impact of adverse emotions on self-efficacy. Recent empirical evidence indicates that mindful awareness is significantly and positively associated with individual self-efficacy (Li and Tong, 2024). When individuals possess high academic self-efficacy, they are more likely to develop confidence in learning, tend to select challenging academic tasks, and persist in academic engagement when encountering difficulties (Aldbyani et al., 2024). Therefore, students with higher levels of mindful awareness may enhance their academic performance through elevated academic self-efficacy.

Meanwhile, conservation of resources theory posits that individuals possess a fundamental motivation to acquire, maintain, and protect resources they value. Initial resource loss often triggers a cascading pattern of further losses, whereas initial resource gain facilitates subsequent resource accumulation (Sonnentag and Meier, 2024). Mindful awareness can be conceptualized as a vital personal characteristic resource that endows individuals with robust psychological regulatory capacity, particularly in emotional management and attentional control (Yavuz Sercekman, 2024). Academic buoyancy, as another personal characteristic resource, specifically refers to students’ adaptive response capacity when confronting everyday academic adversities (Martin and Marsh, 2009). Its core lies in students’ ability to flexibly regulate attention, emotions, and behavioral patterns to cope with ongoing challenges (Martin and Marsh, 2008), thereby achieving rapid recovery from academic setbacks. Consequently, mindful awareness may promote the continuous accumulation of subsequent resources (such as academic buoyancy) by maintaining the sufficiency of initial resources (such as attention and emotion). Recent research demonstrates that mindful awareness significantly and positively influences students’ academic buoyancy (Mohammad Hosseini et al., 2023). Furthermore, studies have shown that mindfulness can significantly affect foreign language achievement through the mediating role of academic buoyancy (Fathi et al., 2025). Therefore, students with higher levels of mindful awareness may enhance their academic performance through elevated academic buoyancy.

Although existing research has confirmed that mindful awareness can influence academic achievement through academic self-efficacy and academic buoyancy respectively, the research subjects have primarily been university students. As a critical period characterized by a significant increase in academic pressure (Steare et al., 2023), the psychological developmental characteristics of junior high school students differ from those of university students, and the applicability of related conclusions to middle school populations warrants examination. Moreover, no studies have yet explored the potential serial mediating roles of academic self-efficacy and academic buoyancy in the process by which mindful awareness influences academic achievement. Resilience theory conceptualizes positive factors that help individuals overcome risks as promotive factors, wherein individual internal factors (such as self-efficacy and self-esteem) are defined as assets, and individual external factors (such as parental support and adult mentors) are defined as resources (Fergus and Zimmerman, 2005). The compensatory model of resilience theory further posits that promotive factors can independently and directly offset the negative effects of risk factors, exerting independent effects on developmental outcomes in the opposite direction to risk factors (Zimmerman, 2013). This suggests that academic self-efficacy, as an individual asset, may exert a positive promotive effect on academic buoyancy by enhancing students’ confidence in coping with academic difficulties and challenges. For instance, Lei et al. (2022), using junior high school students as research subjects, confirmed that academic self-efficacy can significantly influence academic buoyancy; research by Huang and Kou (2025) with university students yielded similar conclusions.

**Figure 1:**
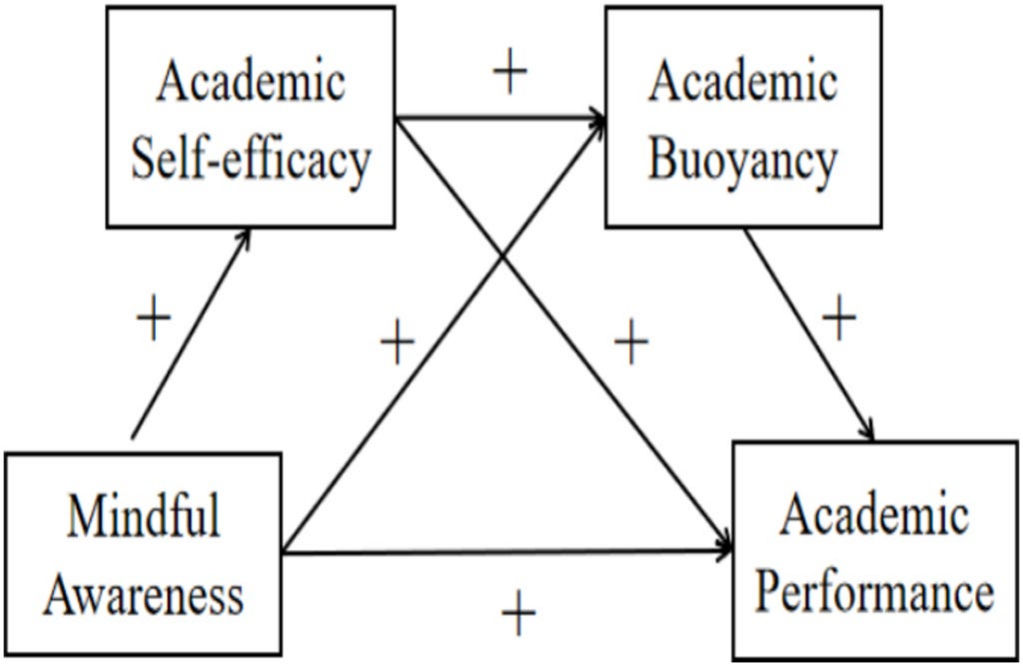
Conceptual model of chain mediation.

In summary, grounded in social cognitive theory, conservation of resources theory, and resilience theory, the present study investigated the mechanism by which mindful awareness influences academic performance through the serial mediating roles of academic self-efficacy and academic buoyancy among Chinese junior high school students. This approach addresses the gap in existing research that has predominantly focused on university student populations and deepens the understanding of the underlying mechanisms through which mindful awareness affects academic performance. Accordingly, the following hypotheses were proposed and a theoretical model was constructed (see Figure 1):

*H1*: Mindful awareness has a significant positive effect on academic performance among junior high school students.

*H2*: Academic self-efficacy mediates the relationship between mindful awareness and academic performance among junior high school students.

*H3*: Academic buoyancy mediates the relationship between mindful awareness and academic performance among junior high school students.

*H4*: Academic self-efficacy and academic buoyancy serially mediate the relationship between mindful awareness and academic performance among junior high school students.

## 2 Materials and methods

### 2.1 Study design and setting

This study employed a cross-sectional self-report questionnaire survey design, targeting Chinese junior high school students and utilizing convenience sampling for data collection. Data collection was conducted from October to December 2023. It should be noted that self-report measures may be subject to social desirability bias and recall bias, and the cross-sectional design limits causal inference.

### 2.2 Participants

This study included a sample of 3,163 adolescents from multiple middle schools across four provinces in China (Yunnan, Zhejiang, Fujian, and Gansu). Demographic analysis revealed a balanced gender distribution, with 1,599 male participants (50.6%) and 1,564 female participants (49.4%). The mean age was 13.3 years (SD = 0.90). Participants were distributed across three grade levels: Grade 7 (*n* = 1,376, 43.4%), Grade 8 (*n* = 1,181, 37.3%), and Grade 9 (*n* = 606, 19.2%). All participants were officially enrolled junior high school students, and questionnaire completion was conducted anonymously.

### 2.3 Measures

#### 2.3.1 Mindful awareness

This study employed the Mindful Attention Awareness Scale to measure participants’ mindful awareness levels. The Mindful Attention Awareness Scale (MAAS) was developed by Brown and Ryan (2003) to assess students’ mindfulness levels and comprises 15 items. Participants were asked to select the most appropriate description for each item based on their actual experiences over the past month (including the current day), using a 6-point Likert scale ranging from “1” (almost always) to “6” (almost never). Higher scores indicate greater levels of present-moment awareness and attention in daily life. This study utilized the Chinese version translated and adapted by Chen et al. (2012), which has demonstrated good reliability and validity in the Chinese cultural context. Confirmatory factor analysis in the present study indicated that the scale had good construct validity (*χ*^2^/df = 13.42, CFI = 0.948, TLI = 0.940, RMSEA = 0.063, SRMR = 0.033) and satisfactory internal consistency reliability α = 0.92.

#### 2.3.2 Academic buoyancy

This study employed the Academic Buoyancy Scale to measure participants’ academic buoyancy levels. The Academic Buoyancy Scale (ABS) was developed by Martin and Marsh (2008) to assess students’ academic resilience and comprises 4 items. A five-point Likert scale was used for scoring (1 = strongly disagree, 2 = disagree, 3 = undecided, 4 = agree, 5 = strongly agree). This study utilized the Chinese version revised by Jia et al. (2020), which was translated and adapted from the original scale and has demonstrated good internal consistency reliability and construct validity. Confirmatory factor analysis in the present study indicated that the scale had acceptable construct validity (*χ*^2^/df = 18.04, CFI = 0.990, TLI = 0.969, SRMR = 0.019, RMSEA = 0.073) and acceptable internal consistency reliability α = 0.78.

#### 2.3.3 Academic self-efficacy

The Academic Self-Efficacy Scale was adapted from the self-efficacy subscale of the Motivated Strategies for Learning Questionnaire (MSLQ) developed by Pintrich and De Groot (1991). The original scale comprises 8 items measured on a 7-point Likert scale ranging from 1 (strongly disagree) to 7 (strongly agree), with higher scores indicating greater academic self-efficacy. The present study employed the Chinese version of this scale. Confirmatory factor analysis (CFA) was first conducted on the original 8-item version to examine its structural validity in the current sample. Results indicated inadequate model fit (*χ*^2^/df = 58.03, CFI = 0.939, TLI = 0.915, RMSEA = 0.122, SRMR = 0.047), with RMSEA substantially exceeding the acceptable threshold of 0.08.

Following best practices in scale adaptation (Boateng et al., 2018), three items were removed based on both statistical evidence and theoretical considerations. Examination of standardized factor loadings revealed that items 1, 2, and 8 exhibited relatively low loadings (ranging from 0.50 to 0.71), whereas the remaining items all demonstrated loadings above 0.78, indicating stronger associations with the latent construct and superior measurement precision. From a theoretical perspective, the removal of these items was justified on the following grounds: (1) Item 1 focused excessively on grade outcomes rather than specific learning capabilities, thus providing a more general and indirect measure of ability beliefs compared to other items; (2) Item 2 was highly redundant with Item 4, with the latter offering more specific and concrete wording; (3) Item 8 was functionally redundant with Item 6 and featured overly verbose expression. The revised 5-item version retained comprehensive coverage of the core dimensions of academic self-efficacy.

The revised 5-item version demonstrated substantially improved model fit (*χ^2^*/df = 17.14, CFI = 0.992, TLI = 0.984, RMSEA = 0.071, SRMR = 0.014), with all indices meeting or exceeding recommended standards. Internal consistency reliability remained high (Cronbach’s α = 0.909 for the revised version vs. α = 0.914 for the original version), and the revised scale exhibited a very strong correlation with the original 8-item scale (*r* = 0.962, *p* < 0.001). Consequently, the revised 5-item scale was adopted for all subsequent analyses in this study.

#### 2.3.4 Academic performance

Class rank serves as a crucial indicator of students’ academic performance. Using large-scale data from Chinese secondary school students, Yu (2020) demonstrated that class rank effectively predicts academic performance. Compared to absolute scores, class rank—based on students’ relative standing within their peer group—controls for variations in teachers’ grading standards, fluctuations in examination difficulty, and other confounding factors, thereby providing a more standardized and comparable assessment metric. Moreover, given that single test performances are susceptible to random errors and may inadequately capture individuals’ true academic capabilities, rank performance across an entire semester exhibits greater stability and consistency, offering a more authentic and comprehensive reflection of students’ academic abilities and sustained effort. From a methodological perspective, large-scale survey research often encounters practical challenges in obtaining standardized test scores, particularly among middle school populations where privacy protection concerns and data access restrictions are more pronounced. Self-reported rank intervals provide a practical alternative that both protects the privacy of adolescent participants and facilitates efficient data collection and analysis. Therefore, the present study employed a self-report measurement approach, whereby participants rated their overall class rank performance over the past semester using a five-point Likert scale: “excellent” (5 points), “good” (4 points), “average” (3 points), “below average” (2 points), and “poor” (1 point). Higher scores indicate higher class rankings and superior academic performance, whereas lower scores reflect lower class rankings and relatively weaker academic performance.

### 2.4 Procedure and ethical considerations

Data collection was conducted through the Wenjuanxing platform, a widely used online data collection tool for scientific research in China, providing a convenient and reliable data collection channel for this study. Given that junior high school students are minors who can only be accessed through schools and guardians, random sampling requires a complete sampling frame but a comprehensive list of all junior high school students nationwide or province-wide was unavailable, and varying levels of school cooperation existed, random sampling could not be implemented. The study employed convenience sampling, with data collection links sent to guardians of target junior high school students via mobile communication. Data collection covered multiple provinces across China, including eastern, central, and western regions with varying levels of economic development, enhancing the geographic and economic diversity of the sample to some extent. Students were eligible to participate in the survey only after obtaining parental informed consent. Participants were explicitly informed that they could freely withdraw from the survey at any time without providing any reason and without any adverse consequences, and all participants agreed to participate in this survey. The entire survey process was conducted anonymously to fully protect participants’ privacy and safety.

To ensure the authenticity and validity of data collection, this study strictly adhered to the following quality control standards: (I) questionnaire respondents must be enrolled students from the target class groups; (II) the Wenjuanxing platform was configured with technical restrictions allowing only one completion per device and IP address to avoid invalid data from duplicate submissions; (III) participants were required to spend a minimum of 300 s completing the questionnaire; (IV) questionnaires showing excessive response consistency patterns across scales were excluded; (V) all participants must be enrolled students registered in the formal education system. The study initially collected 3,800 raw samples, and after systematic data cleaning and quality screening procedures, invalid questionnaires were removed, resulting in a final valid sample size of 3,163 with an effective response rate of 83.24%, meeting the statistical analysis requirements of this study. This study strictly complied with local laws and regulations, the Declaration of Helsinki, and relevant ethical guidelines, and obtained formal approval from the Institutional Review Board of the research institution.

### 2.5 Statistical analysis

First, missing value checks were conducted on all data, with results indicating no missing values. Second, SPSS 25.0 was employed to calculate descriptive statistics for all research variables, including means, standard deviations, skewness and kurtosis coefficients, and Pearson correlation analysis was used to examine the correlations among variables. Subsequently, multiple linear stepwise regression analysis was conducted to perform robustness checks on the hypothesized model. For measurement model validation, AMOS 25.0 was utilized to perform confirmatory factor analysis (CFA) on all research variables to assess the construct validity of the measurement model, and item revisions were made to the Academic Self-Efficacy Scale based on model fit results. Finally, a structural equation model was constructed according to the theoretical framework of the research design, followed by model fit testing and hypothesized path testing. For significance testing of indirect effects, following Hayes’ (2018) recommendations, the Bootstrap resampling method (5,000 samples) was employed to generate 95% bias-corrected confidence intervals. Indirect effects were considered significant when the confidence interval did not include zero.

## 3 Results

### 3.1 Descriptive statistics and correlation analysis

Table 1 presents the means, standard deviations, skewness, kurtosis, and Pearson correlation coefficients among all study variables. First, the absolute values of skewness and kurtosis for all variables were less than 1, indicating that all variables approximated normal distribution following the criteria suggested by Hair et al. (2010) and provided a sound foundation for statistical testing. Second, all scales were significantly and positively correlated with each other (*p* < 0.01), which generally aligned with the correlation expectations proposed in the theoretical framework section of this study.

**Table 1:** Descriptive statistics and correlations among study variables.

Variables	*M*	SD	Skewed	Kurtosis	1	2	3	4
1. Mindful awareness	4.39	0.95	−0.34	−0.19	1			
2. Academic self-efficacy	4.12	1.25	0.13	0.12	0.358^**^	1		
3. Academic buoyancy	3.51	0.76	−0.22	0.05	0.354^**^	0.462^**^	1	
4. Academic performance	3.06	1.07	−0.01	−0.59	0.299^**^	0.327^**^	0.279^**^	1

^**^p < 0.01.

### 3.2 Testing the hypothesized model

To ensure the robustness of the research findings, this study first employed hierarchical regression analysis to conduct preliminary tests of all hypothesized paths. The results of the hierarchical regression are presented in Supplementary material, with the direction and significance of all hypothesized paths being consistent with expectations, providing preliminary support for the subsequent structural equation modeling analysis. Building on this foundation, the study further utilized AMOS 24.0 software to construct a structural equation model to simultaneously test all path relationships and verify the chain mediation effects. After controlling for demographic variables such as gender and grade, the fit indices of the structural equation model demonstrated good performance: *χ*^2^/df = 6.960, CFI = 0.951, TLI = 0.944, RMSEA = 0.043, SRMR = 0.036. All fit indices met acceptable standards, indicating that the hypothesized model fit the data well.

**Figure 2:**
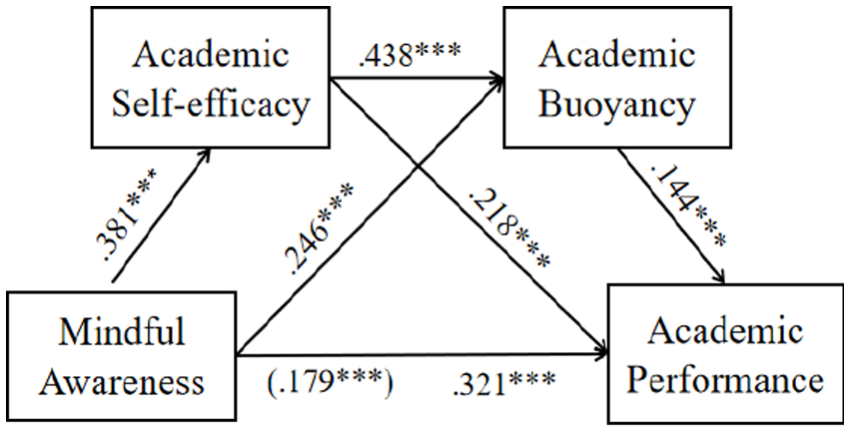
Path coefficients of the structural equation model. *** *p* <.0001. *N* = 3,163. Values outside parentheses are total effects.

Figure 2 presents the results of the direct path tests. The results showed that mindful awareness had significant positive effects on both academic self-efficacy (β = 0.381, *p* < 0.001) and academic buoyancy (β = 0.246, *p* < 0.001). Academic self-efficacy also demonstrated a significant positive effect on academic buoyancy (β = 0.438, *p* < 0.001). Mindful awareness (β = 0.179, *p* < 0.001), academic self-efficacy (β = 0.218, *p* < 0.001), and academic buoyancy (β = 0.144, *p* < 0.001) all exerted significant positive effects on academic performance, thus supporting H1.

Table 2 presents the results of the indirect path and chain mediation path tests. Following the criterion that bias-corrected 95% confidence intervals calculated using the Bootstrap method do not include zero (Hayes, 2018), the significance of the indirect effects was confirmed. Specifically, mindful awareness significantly and positively influenced academic performance through academic self-efficacy (β = 0.083, 95% CI = [0.07, 0.10]), supporting H2. Mindful awareness significantly and positively influenced academic performance through academic buoyancy (β = 0.035, 95% CI = [0.02, 0.05]), supporting H3. Mindful awareness significantly and positively influenced academic performance through the chain mediation of academic self-efficacy and academic buoyancy (β = 0.024, 95% CI = [0.02, 0.03]), supporting H4.

**Table 2:** Indirect effects and their proportions of total effect.

Effects	β	S. E.	95%CI	Ratio (%)^a^
MA → AS→AP	0.083	0.01	[0.07, 0.10]	25.9
MA → AB→AP	0.035	0.01	[0.02, 0.05]	10.9
MA → AS→AB→AP	0.024	0.01	[0.02, 0.03]	7.5
Total indirect effect	0.142	0.01	[0.12, 0.17]	44.2
Total effect	0.321	0.02	[0.29, 0.35]	/

*N* = 3,163. MA, Mindful awareness; AS, Academic self-efficacy; AB, Academic buoyancy; AP, Academic performance; CI, bias-corrected.

Bootstrap sample size = 5,000.

a Ratio = (indirect effect / total effect) × 100%.

In summary, all hypotheses (H1-H4) received empirical support. The effect decomposition results indicated that the mediating effect of mindful awareness on academic performance through academic self-efficacy accounted for 25.9% of the total effect, the mediating effect through academic buoyancy accounted for 10.9% of the total effect, and the chain mediating effect through both variables accounted for 7.5% of the total effect. The three mediating paths collectively explained 44.2% of the total effect, while the direct effect accounted for 55.8%, confirming the validity of the partial mediation model. In terms of the relative strength of the mediating mechanisms, academic self-efficacy served as the primary mediating mechanism, followed by academic buoyancy, while the chain mediating effect of both variables was relatively smaller but still significant.

## 4 Discussion

This study investigated the chain mediating effects of academic self-efficacy and academic buoyancy on the relationship between mindful awareness and academic performance among Chinese junior high school students. The results indicated that mindful awareness can influence academic performance through the mediating roles of academic self-efficacy and academic buoyancy.

### 4.1 Mindful awareness and academic performance

This study found a significant positive correlation between mindful awareness and academic performance among Chinese junior high school students (*r* = 0.299, *p* < 0.01). The standardized regression coefficient of mindful awareness on academic performance also remained significant, demonstrating the cross-cultural universality of the positive effects of mindful awareness. Notably, the correlation coefficient observed in this study is substantially higher than the average effect sizes reported for Western samples in Mao et al.’s (2024) meta-analysis (North America: *r* = 0.112; Europe: *r* = 0.158). This difference may be attributed to the distinct educational contexts in China versus the West. Chinese students face intense academic competition, and academic achievement is highly valued in Chinese culture (Sang et al., 2017). Junior high school students must participate in the high-stakes senior high school entrance examination, which directly determines their eligibility for top-tier high schools and subsequently affects their future college entrance performance and admission opportunities (Cheng and Hamid, 2025). In such a high-risk environment, improvements in academic performance often come at the cost of increased psychological stress, making the buffering effect of mindful awareness as a psychological resource especially salient. In contrast, Western educational evaluation systems are more diversified, with flexible pathways for academic advancement and relatively lower pressure regarding academic performance (Han, 2025). As a result, the demand for psychological resources is comparatively reduced, and the protective effect of mindful awareness is less pronounced. Academic performance in Western contexts may be more influenced by external environmental factors such as social relationships and personal abilities.

### 4.2 The independent mediating roles of academic self-efficacy and academic buoyancy

This study found that mindful awareness exerts a significant positive influence on junior high school students’ academic performance through two separate pathways: academic self-efficacy and academic buoyancy. This finding aligns with the perspectives of social cognitive theory (Scott et al., 2024) and conservation of resources theory (Halbesleben et al., 2014). The non-judgmental and decentered qualities of mindful awareness can enhance individuals’ emotional regulation abilities and foster the replenishment of positive psychological resources, effectively buffering the stress, difficulties, and challenges encountered in academic life. In turn, this process promotes academic self-efficacy and academic buoyancy, ultimately contributing to improved academic performance.

However, our findings indicate that academic self-efficacy serves as the primary mediator (β = 0.083), with a stronger effect than academic buoyancy (β = 0.035). This may be because academic self-efficacy, as a relatively stable core cognitive resource, applies across all academic contexts and has a direct impact on the setting of academic goals, the level of effort exerted, and the perseverance displayed during academic tasks (Scott et al., 2024). By contrast, academic buoyancy is oriented toward coping with everyday minor setbacks, characterized by greater contextual specificity and immediate reactive features (Martin and Marsh, 2009), resulting in higher volatility and context dependency.

Moreover, mindful awareness primarily operates through metacognitive awareness and emotional regulation mechanisms, while the formation of academic self-efficacy depends heavily on cognitive appraisal and emotional arousal—mechanisms closely aligned with those of mindful awareness at both the cognitive and emotional levels. Large-scale international meta-analyses have also shown that self-efficacy is the strongest predictor of academic achievement, exhibiting the largest effect size of all psychological variables and underscoring its foundational role in influencing academic performance (Richardson et al., 2012). Existing research further suggests that shared variance between mediator variables in multivariate structural models may suppress their respective independent contributions (Putwain et al., 2020), which could partially explain the attenuated effect of academic buoyancy on academic performance.

### 4.3 The chain mediating role of academic self-efficacy and academic buoyancy

This study confirmed the chain mediating role of academic self-efficacy and academic buoyancy in the relationship between mindful awareness and academic performance among Chinese junior high school students, which holds significant theoretical and practical implications. The theoretical mechanism through which mindful awareness influences academic performance was further elaborated, providing a foundation for future research in this domain. Academic self-efficacy was found to exert a significant positive effect on students’ academic buoyancy, consistent with the compensatory model described in resilience theory (Zimmerman, 2013). As a promotive factor, academic self-efficacy enables students to cope with everyday academic difficulties by compensating for the depletion of psychological resources that occurs when facing academic setbacks. Therefore, students with higher levels of mindful awareness possess stronger emotional regulation abilities, which are associated with greater academic self-confidence in learning contexts. This increased confidence further enhances their academic self-efficacy, motivating them to proactively engage in challenging academic tasks and facilitating quicker recovery from setbacks encountered in the academic process. These findings provide an important practical intervention pathway for secondary schools aiming to enhance junior high school students’ academic self-efficacy and academic buoyancy, thereby promoting improvements in academic performance.

## 5 Conclusion

Based on social cognitive theory, conservation of resources theory, and resilience theory, this study constructed a chain mediation model to systematically reveal the internal mechanism through which mindfulness awareness influences academic performance among Chinese middle school students via academic self-efficacy and academic buoyancy. The findings indicated that mindfulness awareness exerted significant positive effects on academic performance through both the independent mediation of academic self-efficacy and academic buoyancy, as well as through their chain mediation, with academic self-efficacy playing the primary mediating role. This study verified the important role of mindfulness awareness in promoting positive psychological qualities among Chinese middle school students, providing educators and relevant practitioners with a crucial theoretical foundation and intervention pathway for enhancing students’ academic performance. School administrators can incorporate brief mindfulness meditation training (e.g., 2–4 sessions per week, 10–15 min each) into the curriculum to fully leverage the role of mindfulness awareness in building students’ psychological resources. Teachers can integrate simple mindfulness practices into daily instruction (e.g., 2–3 min of mindful breathing before class) to cultivate students’ academic self-efficacy and academic buoyancy, thereby effectively promoting academic performance. In conclusion, cultivating middle school students’ mindfulness awareness level represents a cost-effective and efficient positive psychological intervention strategy, offering a feasible and effective pathway for enhancing the academic performance of Chinese middle school students.

## 6 Limitations

This study has several limitations. First, the cross-sectional design precludes causal inferences among the pathways in the serial mediation model, and convenience sampling may introduce self-selection bias. Future research should employ longitudinal designs or time-series analyses to better establish causal relationships among variables, while adopting more rigorous random sampling methods to reduce sample bias. Second, due to China’s “Double Reduction” policy, most schools no longer disclose students’ grades publicly. Consequently, this study measured academic performance through self-reported class rankings, which may be subject to social desirability bias and recall bias, thereby limiting measurement objectivity. Future research should incorporate multiple assessment methods, including objective academic indicators (e.g., standardized test scores), teacher evaluations, and peer assessments, to enhance measurement reliability and validity. Third, significant between-class differences in academic performance and clustering effects may exist. Future research should construct multilevel linear models or employ class-level random effects models to control for class-level variance and improve the accuracy of statistical inference. Fourth, although the relationships among individual variables in this study have been validated in Western research, the serial mediation model (mindfulness → academic self-efficacy → academic buoyancy → academic performance) was tested exclusively among Chinese junior high school students. Chinese collectivist culture places strong emphasis on academic performance as the primary criterion for evaluating students, whereas Western individualist cultures employ more diversified evaluation systems. These cultural differences may influence the strength of the mediating roles of academic self-efficacy and academic buoyancy. Therefore, future research should validate the cross-cultural applicability of this model among Western middle school student samples.

## Data Availability

The data that support the findings of this study are available from the corresponding author upon reasonable request.
