# Supplementary material for: How does mindful awareness impact academic performance in junior high school students? A chain mediation effect based on academic self-efficacy and academic buoyancy
Source: Front Psychol. 2025 Nov 11;16:1687223. doi: 10.3389/fpsyg.2025.1687223 (PMC12643856; doi:10.3389/fpsyg.2025.1687223)
Supplement: Supplementary file 1 [file Supplementary_file_1.zip › Appendix A/Results of Stepwise Regression for the Chain Mediation Model.docx]

Results of Stepwise Regression for the Chain Mediation Model

| Variables | AS | | AB | | AP | | |
| --- | --- | --- | --- | --- | --- | --- | --- |
|  | M1 | M2 | M3 | M4 | M5 | M6 | M7 |
| Controlled variables |  | | | | | | |
| Gender | -.148^***^ | -.135^***^ | -.127^***^ | -.063^***^ | .096^***^ | .144^***^ | .152^***^ |
| Age | .015 | .028 | -.002 | .000 | .030 | .033 | .033 |
| Grade | -.067^*^ | -.065^*^ | -.007 | .019 | -.047 | -.027 | -.030 |
| Independent variables |  | | | | | | |
| MA |  | .352^***^ |  | .218^***^ |  | .206^***^ | .178^***^ |
| Mediating variables |  |  |  |  |  |  |  |
| AS |  |  |  | .375^***^ |  | .274^***^ | .225^***^ |
| AB |  |  |  |  |  |  | .131^***^ |
| R^2^ | .026 | .129 | .016 | .258 | .010 | .165 | .178 |
| ΔR^2^ | .025 | .148 | .015 | .257 | .009 | .164 | .176 |
| *F* | 27.97^***^ | 138.41^***^ | 17.32^***^ | 220.05^***^ | 10.31^***^ | 124.97^***^ | 113.92^***^ |
| Note.N = 3,163. MA=Mindful Awareness;AS=Academic Self-efficacy;AB=Academic Buoyancy; | | | | | | | |
| AP=Academic Performance; | | | | | | | |
| **P*<.05 ***p*<.001 | | | | | | | |
